# A methodological review protocol of the use of Bayesian factor analysis in primary care research

**DOI:** 10.1186/s13643-020-01565-6

**Published:** 2021-01-08

**Authors:** Hao Zhang, Tibor Schuster

**Affiliations:** Department of Family Medicine, McGill University, 5858 Chemin de la Côte-des-Neiges, Suite 300, Montreal, QC H3S 1Z1 Canada

**Keywords:** Bayesian, Factor analysis, Primary care, Family medicine

## Abstract

**Background:**

The development of questionnaires for primary care practice and research is of increasing interest in the literature. In settings where valuable prior knowledge or preliminary data is available, Bayesian factor analysis can be used to incorporate such information when conducting questionnaire construct validation. This protocol outlines a methodological review that will summarize evidence on the current use of Bayesian factor analysis in the primary care literature.

**Methods:**

A comprehensive search strategy has been developed and will be used to identify relevant literature (research studies in primary care) indexed in MEDLINE, Scopus, EMBASE, CINAHL, and Cochrane Library. The search strategy includes terms and synonyms for Bayesian factor analysis and primary care. The reference lists of relevant articles being identified will be screened to find further relevant studies. At least two reviewers will independently extract data and resolve discrepancies through consensus. Descriptive analyses will summarize the use and reporting of Bayesian factor analysis approaches for validating questionnaires applicable to primary care.

**Discussion:**

This methodological review will provide a comprehensive overview of the current use and reporting of Bayesian factor analysis in primary care and will provide recommendations for its proper future use.

**Systematic review registration:**

PROSPERO CRD42018114978

## Background

In the past decades, there has been a proliferation of primary care research studies with publications in the field increasing by about 75% and major primary care research databases approximately tripling the amount of information stored between 2004 and 2013, just in the UK [1, 2]. Despite this noticeable growth of available health information in various primary care domains, ensuring adequate quality of the data being collected and processed remains a major challenge [3, 4]. In the context of primary care practice and research, validation of questionnaire instruments is critical for the development of reliable measurement tools that help informing day-to-day clinical decision-making and evidence-based medicine.

Factor analysis examines the strength of correlation of each individual questionnaire item with respect to a set of latent domains or constructs (i.e., the “factors”) and is widely used for questionnaire construct validation in education, psychological, social, and healthcare research [5]. The empirical validation of measurement properties of an instrument through factor analysis typically involves a relatively large sample of completed questionnaires. The suggested minimum sample size for conventional confirmatory factor analysis in the literature ranges between approximately 100 and 300 responses [6]. An additional limiting element is that individuals who participate in pilot validation studies can typically not be involved in subsequent phases of the questionnaire instrument validation. This is critical as primary care research often applies in community settings and may be aimed at addressing the needs of populations that are comparatively low in numbers and difficult to identify and recruit.

Bayesian methods offer promising solutions to this impasse as the incorporation of prior information can increase efficiency in estimating target parameters compared to conventional methods [6–9]. Bayesian factor analysis for instrument development enables the inclusion of knowledge and opinions of health professionals, patients, and other stakeholders, potentially increasing the practical value and applicability of the instrument.

Nevertheless, when screening the primary care literature for questionnaire validation studies, Bayesian factor analysis appears to be underutilized. Furthermore, the implementation of the Bayesian approach and reporting of findings seem to vary largely across studies. To our best knowledge, no methodological review has yet been undertaken to quantify and qualify the use and reporting of Bayesian factor analysis in primary care. Therefore, the aim is to provide the first comprehensive methodological review on this matter.

The objectives of the methodological review will be (1) to identify and consolidate the existing Bayesian factor analysis approaches in primary care practice and research settings, (2) to assess the quality of the implementation and reporting of Bayesian factor in these settings, and (3) to summarize the used approaches for prior elicitation and Bayesian inference, including different estimation procedures and software routines.

## Methods/Study design

### Search methods for identification of studies

A comprehensive search strategy with high sensitivity will be adopted to identify all potential records relevant to the field of primary care and family medicine as previously described [10, 11]. The search strategy includes the terms and synonyms for Bayesian factor analysis and primary care as shown in Table 1. The search strategy is developed with a specialized librarian and will be conducted by at least two reviewers independently. Searches of electronic databases with hand searches of reference lists will be combined. The computer-based searches will combine medical subject headings (MeSH) terms, free text, and full text.

**Table 1:** MEDLINE search strategy through the PubMed interface

Concepts	PubMed
Bayesian	(bayes*[tw] OR “Bayes Theorem”[Mesh] OR Gibbs sampler OR “MCMC” OR “prior distribution” OR “posterior distribution”) AND
Factor Analysis	(“Factor Analysis, Statistical”[Mesh] OR factor analys*[tw] OR item domain correlation* OR item response theor* OR structure equation model* OR latent variable* OR factor loading*) AND
Primary Care	(“family”[all fields] OR physician*[all fields] OR practice*[tw] OR “primary care”[all fields] OR “Primary Health Care”[mh] OR primary[tw] OR general pract*[tiab] OR gp[tiab] OR gps[tiab] OR clinic*[tiab] OR refer*[tiab] OR visit*[tiab] OR outpatient*[tiab] OR consult*[tiab] OR communit*[tiab] OR ambulatory[tiab] OR centre*[tiab] OR office[tiab])

#### Databases and time frame

Articles from MEDLINE, Embase, Cochrane Library, CINAHL, and Scopus will be identified. All relevant articles published before January 1, 2020, will be considered.

#### Searching other resources

Google Scholar will be manually scanned for the first 200 to 300 records for supplementary information [12]. Reference lists and the future citation of the retrieved articles will be manually searched with two additional rounds. As currently no well-established guidelines exist on conducting methodological reviews, we will follow the general recommendations in the literature [13], the guidelines under development [14], and the PRISMA-P statement for the methodological review [15, 16]. Review of review articles will serve for identifying the literature covered within the reviews.

#### Types of studies

Quantitative and empirical research studies, methodological studies using Bayesian factor analysis, review articles, conference abstracts, and thesis or dissertation documents will be included. Research studies using similar model structures such as structural equation models and latent variable models, as well as item response theory, factor loadings, and item domain correlations, will be included. Some conferences publish full-text papers, i.e., conference proceedings alongside with abstracts. Only conference abstracts with respective full-text access will be considered in the methodological review.

#### Inclusion criteria

The inclusion criteria of the review require literature to match the following three themes: “in the context of primary care practice,” “Bayesian methods,” and “factor analysis.” The definition of primary care follows that of the American Academy of Family Physicians as being “comprehensive,” “first contact,” and “continuing”; meanwhile, it covers “any undiagnosed sign, symptom, or health concern” [17]. The term “Bayesian methods” refers to any inferential method that employs prior distributions in conjunction with observed information to arrive at an estimate for the parameter of interest.

#### Exclusion criteria

Editorials, commentaries, book reviews, hypotheses, critical appraisals, reflections, surveys, case reports or studies, or studies that do not employ Bayesian methods will be excluded. Studies that include some of the keywords but use them under different connotations or references will be excluded. Examples of ineligible use of keywords include “primary studies,” “prior to,” “human epidermal growth factor,” and “genetic factor.”

Bayesian methods used in other types of analyses, such as Bayes rule, Bayes or Bayesian factor studies, variational Bayes, Bayesian Information Criterion/Criteria, Bayesian random effects models, Bayesian/Bayes network, belief network, and Bayes(ian) model or probabilistic directed acyclic graphical model will be excluded. Studies not in family medicine or primary care but using related terminology will be excluded. Examples are “a family of methods” and “exponential family.”

### Data collection and analysis

#### Selection of studies

Titles and abstracts of studies will be sequentially screened using the search strategy by at least two independent reviewers using the software Rayyan [18]. If no information is given in the title or abstract about any of the three inclusion criteria, i.e., no indication about whether the study is applying Bayesian methods, using factor analysis, or in primary care, those studies will be included at the initial stage of screening. In indecisive situations, for example, when the term “factor analysis” is mentioned but not specified whether it is Bayesian or not Bayesian, the article will be kept for the next round of full-text review. The full text of articles that meet the inclusion criteria will be retrieved and examined independently by *k>2* reviewers, each reviewing one out of the *k* portions of the identified articles that are randomly assigned to each reviewer. All articles will be also reviewed by the main author. Any disagreement between the reviewers and the main author about the eligibility of specific studies will be discussed, and an additional reviewer will be involved if necessary, until a consensus is reached. For studies with multiple publication records, the most comprehensive or up-to-date record will be used.

#### Data extraction and management

Data extraction and data preparation will be facilitated using Microsoft Excel and the statistical software package R. All records will be coded and categorized under the predefined themes in the codebook from the Canadian Institute of Health Research (CIHR) grants and rewards guide [19]. Despite existent guidelines and recommendations on reporting of general Bayesian methods, confirmatory factor analysis, and questionnaire development, no single comprehensive recommendation was found on the reporting of Bayesian confirmatory factor analysis [20–22]. Where applicable, the following data will be extracted: type of journal, publication date, geographical location, sample size, number of items or questions used for the Bayesian factor analysis, number of factors, domains or constructs, reported item-domain correlations, regression parameters, factor loadings, parameters of structural equation models, use of prior information and assumed prior distributions, and the primary care settings. A standardized predesigned data collection form will be used for data extraction. The assessment criteria below will be followed:

1. Did the authors use either Bayesian confirmatory factor analysis or Bayesian exploratory factor analysis or Bayesian latent variable model or Bayesian structure equation modeling?
2. If they used (at least) one of the listed methods, what was the parameter of interest they were aiming to estimate: item-to-domain correlation, factor loading, or latent model regression parameter? In other words, for which parameter did they impose a prior distribution?
3. How did investigators inform their prior distribution of the respective parameter? What was the prevalence of studies that employed non-informative priors?
4. If they mention the term “factor loading,” did they explain it, and if, how did they interpret it, i.e., as an item-to-domain correlation or as a model parameter (latent variable regression coefficient)?
5. Did they report standardized factor loadings or parameter estimates that exceeded an interval of [ -1, 1]?
6. Were credible intervals or confidence intervals reported for factor loadings, item-to-domain correlations, model parameters, or regression coefficients?
7. What software or libraries were used? Were software codes or original data made available? (reproducibility)

The data extraction form used to summarize information obtained from the identified articles will be pilot tested to identify possible sources of error or imprecision. For this purpose, all reviewers involved will extract the data from a selected set of articles using the data extraction form. The extracted data will then be compared and sources for potential mismatches or errors discussed and resolved.

#### Assessment of quality of implementation and reporting of Bayesian methods

The risk of bias in individual studies is not applicable to and will not be assessed in the review since the goal is to summarize the use and reporting of Bayesian questionnaire validation methods, i.e., there is no single effect parameter that is of primary interest. The data collected across studies will indicate the presence or absence of each of the seven criteria for assessing the appropriateness of design, conduct, and reporting. The quality of implementation and reporting of Bayesian methods for each eligible study will be assessed and rated on an ordinal scale with the following levels: very low, low, moderate, and high on the following aspects: reporting about methodology, Bayesian model, estimated parameters, prior elicitation, and basic contextual information provided. The quality assessment will be conducted independently by two expert statisticians (H.Z. and T.S.) and presented in tables in the final publication of the methodological review. No available critical tools exist to appraise the use of Bayesian factor analysis; however, the proposed quality appraisal (i.e., a methodological “peer review”) by the authors will help to identify prevalent issues and initiate discussions of better reporting standards.

### Strategy for data descriptions and synthesis

A descriptive synthesis of the findings from the included studies with graphs and tables will be provided detailing the use of Bayesian factor analysis based on a common analytical framework on authors, years of publication, estimates, the number of publications over time, geographical locations, the study populations, the aims of the study, data types, key information about the data (e.g., sample sizes, number of questions in a questionnaire, or number of domains or factors), the type of Bayesian method used, and different estimation procedures and software routines (e.g., analytical solutions vs. sampling-based solutions).

## Anticipated results

### Description of studies

The current use of Bayesian factor analysis will be summarized through descriptive statistics, for example, frequency distributions displaying the prevalence of the seven predefined assessment criteria across studies. A subjective quality appraisal developed in this review will be useful in initiating discussions of better reporting standards based on the review.

## Discussion

This methodological review will provide a detailed summary of how Bayesian factor analysis methods are applied in primary care practice and research settings. It will enable the identification of shortcomings in the application and reporting of Bayesian factor analysis studies within the context of primary care and will help to improve practice through discussing and refining current reporting standards. No one single agreed definition of the research domain of primary care and family medicine yet exists, which might affect the search results. Another weakness is the lack of a standard appraisal instrument for assessing the appropriateness of design, conduct, and reporting of Bayesian factor analysis. However, the quality appraisal conducted by the authors will be helpful in identifying major gaps and will potentially inform the future development of such an appraisal tool.

## Data Availability

Not applicable

## Abbreviations

CINAHL: Cumulative Index to Nursing & Allied Health Literature
EMBASE: Excerpta Medica database
UK: United Kingdom
PRISMA: Preferred Reporting Items for Systematic Reviews and Meta-Analyses
